# Molecular structure and evolution mechanism of two populations of double minutes in human colorectal cancer cells

**DOI:** 10.1111/jcmm.16035

**Published:** 2020-10-30

**Authors:** Xueyuan Jia, Rongwei Guan, Xiaobo Cui, Jing Zhu, Peng Liu, Ling Zhang, Dong Wang, Yang Zhang, Kexian Dong, Jie Wu, Wei Ji, Guohua Ji, Jing Bai, Jingcui Yu, Yang Yu, Wenjing Sun, Feng Zhang, Songbin Fu

**Affiliations:** ^1^ Laboratory of Medical Genetics Harbin Medical University Harbin China; ^2^ Key Laboratory of Preservation of Human Genetic Resources and Disease Control in China Ministry of Education Harbin Medical University Harbin China; ^3^ Obstetrics and Gynecology Hospital State Key Laboratory of Genetic Engineering at School of Life Sciences Institute of Reproduction and Development Fudan University Shanghai China; ^4^ Key Laboratory of Reproduction Regulation of NPFPC Collaborative Innovation Center of Genetics and Development Fudan University Shanghai China; ^5^ Scientific Research Centre the Second Affiliated Hospital of Harbin Medical University Harbin China

**Keywords:** colorectal adenocarcinoma, double minutes, evolution mechanism, extrachromosomal DNA, gene amplification, molecular structure

## Abstract

Gene amplification chiefly manifests as homogeneously stained regions (HSRs) or double minutes (DMs) in cytogenetically and extrachromosomal DNA (ecDNA) in molecular genetics. Evidence suggests that gene amplification is becoming a hotspot for cancer research, which may be a new treatment strategy for cancer. DMs usually carry oncogenes or chemoresistant genes that are associated with cancer progression, occurrence and prognosis. Defining the molecular structure of DMs will facilitate understanding of the molecular mechanism of tumorigenesis. In this study, we re‐identified the origin and integral sequence of DMs in human colorectal adenocarcinoma cell line NCI‐H716 by genetic mapping and sequencing strategy, employing high‐resolution array‐based comparative genomic hybridization, high‐throughput sequencing, multiplex‐fluorescence in situ hybridization and chromosome walking techniques. We identified two distinct populations of DMs in NCI‐H716, confirming their heterogeneity in cancer cells, and managed to construct their molecular structure, which were not investigated before. Research evidence of amplicons distribution in two different populations of DMs suggested that a multi‐step evolutionary model could fit the module of DM genesis better in NCI‐H716 cell line. In conclusion, our data implicated that DMs play a very important role in cancer progression and further investigation is necessary to uncover the role of the DMs.

## 1 INTRODUCTION

Gene amplification, as a common feature of genomic instability in many tumours, is highly associated with tumorigenesis and chemoresistance. Cytogenetically, gene amplification chiefly manifests as homogeneously stained regions (HSRs) or double minutes (DMs).^1^, ^2^ In 2017, the term extrachromosomal DNA (ecDNA) was first proposed for extrachromosomal chromatins at molecular level.^3^ No matter cytogenetic DMs and molecular ecDNA, they usually carry amplification of oncogenes or drug‐resistant genes in cancer. DMs and ecDNA were regarded as diagnostic markers in clinical practice to monitor cancer progression, occurrence, and prognosis.^4^, ^5^, ^6^ A recent survey of a compendium of cancer cells and cell lines in Glioblastoma Multiforme (GBM) provides direct evidence that extrachromosomal amplification of oncogenic elements enhances genomic diversity during cancer evolution.^3^ The farther research showed how ecDNA elements could mark major clonal expansions in otherwise‐stable genomic backgrounds and related ecDNA presence to cancer progression and also pointed out that ‘whether ecDNA size and structure affect the mechanism of tumorigenesis is unclear and is another reflection of the lack of knowledge of extrachromosomal DNA, in particular as an understudied domain in cancer’.^7^ However, possible mechanisms and contributions of DMs/ecDNA for cancer progression are still unknown.

Currently, it has been documented that extrachromosomal chromatins arose from circularization of co‐amplified DNA fragments from multiple chromosomal loci.^8^, ^9^ In earlier studies, researchers have attempted to measure the complex genomic rearrangements of the amplicons in cancer by array comparative genomic hybridization (CGH), fluorescence in situ hybridization (FISH), quantitative PCR and more advanced techniques such as whole genome sequencing.^10^, ^11^, ^12^ The rejoining patterns of the amplicons which constitute DMs have been revealed accordingly.^13^, ^14^, ^15^ Gibaud's study supported that break‐fusion‐bridge cycles and/or chromosome fragmentation, which mainly involved non‐homologous end joining (NHEJ) to induce the reconnection of chromosomal breakage, may drive the complex structures of intrachromosomal amplifications.^14^ Another study by Gibaud's team found that the formation of DMs may be accomplished through V(D)J‐like illegitimate recombination.^15^ Stolazzi and colleagues confirmed the episome model, suggesting that DMs were formed by amplification, excision and fusion junctions mediated by NHEJ.^13^ These data suggested that concept of genesis and structure of DMs are controversy and multiple models have been proposed according to their observations. Recently, combinational usage of whole genome sequencing and FISH analysis has led us into the primary understanding of DM structures in glioblastoma multiforme,^16^ solid tumours or cancer cell lines.^17^, ^18^ Analysing tool is being developed, and it would get more precise resolution of extrachromosomal chromatins from high‐throughput sequencing data. It would permit a deeper understanding of the scale, scope and contents of extrachromosomal chromatins in cancer and their association with clinical features.^6^

Because of the diversity of extrachromosomal chromatins in size, amount, molecular composition and organization in single or multiple cells,^10^ it is important to identify the molecular architecture of extrachromosomal chromatins. In ecDNA study, Paul's team used three cell lines and constructed partial molecular architecture at genome level.^19^ Nevertheless, due to the heterogeneity of DMs/ecDNA, without co‐localization analyses of the different amplified regions at the cellular level (Multiplex‐fluorescence in situ hybridization, M‐FISH), the reconstructed DMs/ecDNA structures may not be accurate or complete. More important, according to the research, heterogeneity of extrachromosomal chromatins was dynamic during drug resistance development and cell proliferation. Resistance to doxorubicin (DOX) and methotrexate (MTX) in human osteosarcoma cells was a multigenic process involving both gene copy number and expression changes.^20^ In addition to changes in the copy number of the gene amplification, its form of existence may also change. Analysing of amplification in GBM39 cells, the ecDNA reintegrated as HSRs after erlotinib treatment.^3^ On removal of the treatment, the ecDNA amplicons re‐emerged.^21^ Through these studies, it helps us to consider that accelerated heterogeneity of cancer cells through DMs/ecDNA may increase the likelihood of tumorigenesis and chemoresistance.

The human colorectal adenocarcinoma cell line NCI‐H716 carries DMs, and the molecular structure of them has not been explicitly defined.^10^ In the present study, we employed new techniques to re‐identify the subpopulations in order to visualize DM formation and their molecular structure in NCI‐H716 cells. As a result, we built a framework for characterizing the molecular structure of DMs with integrated analyses including M‐FISH, high‐resolution array CGH, NimbleGen capture array, PacBio RS DNA system, Illumina HiSeq X Ten platform and chromosome walking. Our data implicated that both NHEJ/ microhomology‐mediated end joining (MMEJ) and fork stalling and template switching (FoSTeS)/ microhomology‐mediated break‐induced replication (MMBIR) mechanisms, in a multi‐step evolutionary way, illustrated the rearrangements during DM formation in NCI‐H716 cells.

## 2 MATERIALS AND METHODS

### 2.1 Cell culture

The human colorectal cancer cell line NCI‐H716 with spontaneous DMs was purchased from American Type Culture Collection (ATCC, VA, USA). The cells were cultured in RPMI‐1640 medium (Invitrogen, CA, USA) supplemented with 10% foetal bovine serum (Invitrogen) in a humidified atmosphere of 5% CO_2_ at 37°C. Cells were authenticated by short tandem repeat profiling analysis (Beijing Microread Genetics, Beijing, China).

### 2.2 M‐FISH analysis

Metaphase chromosomes of the NCI‐H716 cells were prepared as previously described.^22^ The slides were either stained with Giemsa (Sigma‐Aldrich, MO, USA) or stored at room temperature for M‐FISH analysis. Bacterial artificial chromosome (BAC) clones were purchased from the BACPAC Resources Center (Children's Hospital Oakland, CA, USA) (Table S1). BACs clones specific to amplified regions were selected as probes and labelled by random primers with cy5‐dUTP, cy3‐dUTP (GE Healthcare, Little Chalfont, UK), and Green‐dUTP (Enzo Life Sciences, NY, USA). All probes were hybridized with metaphase spreads,^22^ which were counterstained with 4,6‐diamidino‐2‐phenylindole (DAPI). Images were captured using fluorescence microscope (Leica, Wetzlar, Germany) and analysed with the MetaMorph Imaging System (Universal Imaging Corp, NY, USA).

### 2.3 High‐resolution array CGH

DNA processing, microarray handling and data analysis were performed according to the protocol by manufacturer (Agilent Oligonucleotide Array‐Based CGH for Genomic DNA Analysis, version 6.1, August 2009, Agilent Technologies, CA, USA) with minor modifications. Oligonucleotide‐based Human Genome Microarrays (Agilent Technologies) containing 60 K, 180 K, 400 K and 1 M features were used for hybridization, among which 2 × 400 K format was used to examine genome‐wide copy number alteration (Figure S1). The 4 × 180 K format targeting amplified regions was customized microarray that we created by using the Agilent eArray online system (https://earray.chem.agilent.com/) for interrogating the genome of DMs with high‐resolution and accuracy. Data were quality controlled and extracted using Feature Extraction (version 9.1, Agilent Technologies, CA, USA), and subsequently analysed by Agilent Genomic Workbench (version 7.0, Agilent Technologies, CA, USA).

### 2.4 DNA Hybrid Capture and PacBio RS sequencing

Genomic coordinates of DMs were provided by high‐resolution array‐CGH analysis. Customized NimbleGen 2.1M sequence capture arrays were fabricated using SeqCap v2 software. The captured library preparation and NimbleGen Sequence Capture Arrays were performed according to the manufacturer's protocol, which was adapted from the company's application notes (Roche NimbleGen, WI, USA). The captured DNAs were generated by ligating PacBio SMRTbell™ adapters to both ends of linear DNA fragments. These DNA fragments were sequenced on the PacBio RS as a continuous circle. Then, clear data were mapped onto reference human genome (GRCh37/hg19) using Burrows‐Wheeler Aligner (BWA) to filter out the reads without SVs. Subsequently, all the identified fusion sequences were also verified with the BLAST‐Like Alignment Tool (BLAT) and Basic Local Alignment Search Tool (BLAST).

### 2.5 Whole genome sequencing

Genomic DNA of the NCI‐H716 cells was extracted, quantified and purified with HiSeq X Ten protocol (Illumina, CA, USA). DNA fragments were ligated with adaptor oligonucleotides to form paired‐end DNA libraries with an insert nucleotide of 500 bp. This library was amplified by PCR with adaptor‐specific primers and sequenced by Illumina HiSeq X Ten instrument to obtain up to ∼150 million reads (Novogene, Beijing, China). Reads that aligned to genomic regions were collected for mutation identification and subsequent analysis. Samtools mpileup and bcftools were used to do variant calling and identify single nucleotide polymorphisms (SNPs), insertions and deletions (INDELs). Control‐FREEC was utilized to do copy number variations (CNVs) detection. And BreakDancer was applied to detect SV information.

### 2.6 Chromosome walking

Sequences of Junction VII and Junction VIII were acquired using GenomeWalker universal kit and Advantage 2 PCR Kit (Clontech Laboratories Inc, CA, USA) according to the manufacturer's instructions. Normal human DNA was used as control. All the junction sequences were validated by Sanger sequencing.

## 3 RESULTS

### 3.1 DMs in NCI‐H716 cells were comprised of multiple highly amplified regions from chromosome 8q24.12‐21 and 10q26.13

**Figure 1:**
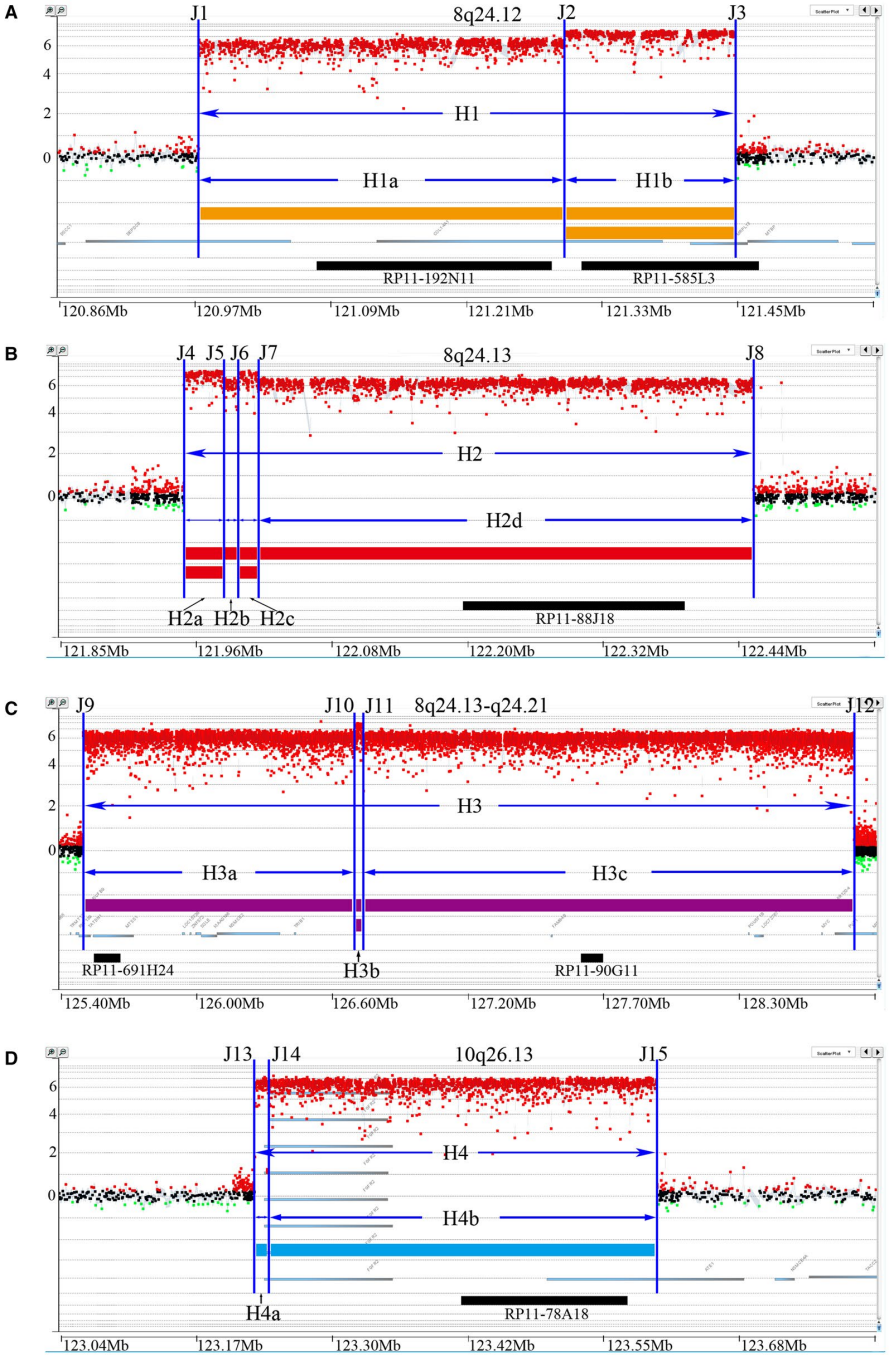
Four amplified regions were determined in NCI‐H716 cells with an Agilent array‐CGH chip with high‐density probes. The yellow strip in A, red strip in B, purple strip in C and blue strip in D represented each corresponding amplification region—H1, H2, H3 and H4. The majority of the copy numbers were around 2^6^, shown with single line. The majority of the copy numbers were around 2^7^, shown with double lines. X‐axis represented chromosome coordinates. Y‐axis represented log_2_ ratios of the copy number normalized by normal controls, showing distinct sub‐regions with different overall copy numbers. Blue vertical lines depict boundary positions for each amplicon. The position of the BACs was marked with black strip

In order to clarify the origin of DMs, we first used metaphase chromosome analysis to confirm the existence of DMs, and observed large amounts of DMs in NCI‐H716 cells. Then, to obtain the amplified regions, an Agilent 2 × 400 K human genome CGH microarray was applied to examine the CNV. We identified four major amplified regions originating from 8q24.12‐21 and 10q26.13 in NCI‐H716 cells. The amplicons were named as Amplicon H1, H2, H3 (from centromere to telomere) on chromosome 8q24.12‐21 and Amplicon H4 on 10q26.13 (Figure S1). Based on CGH microarray results, we designed a customized high‐resolution microarray (mean distance ≈ 200 bp in the amplified regions). Through detailed analysis of microarray results, we found that amplicons H1, H2, H3 and H4 were composed of several sub‐amplicons depending on different amplification levels. These sub‐amplicons were therefore named Amplicon H1a, H1b, H2a, H2b, H2c, H2d, H3a, H3b, H3c, H4a and H4b (Figure 1). In the four major amplicons, the majority of the copy numbers were as high as 2^6^. However, a dramatically higher amplification level was observed in sub‐amplicons (H1b, H2a, H2c, and H3b), twice of the major amplicons (Figure 1). Notably, a high density for probing was required for accurate detecting of subtle structure variations. It might be the reason that why we had not been able to capture the subtle changes in DNA copy number in the amplification regions of NCI‐H716 cells in previous studies. These results suggested that DMs in NCI‐H716 cells containing highly amplified sequence with different copy number derived from chromosome 8q24.12‐21 and 10q26.13.

### 3.2 DMs were heterogeneously organized defined by localization of amplicons in NCI‐H716 cells

**Figure 2:**
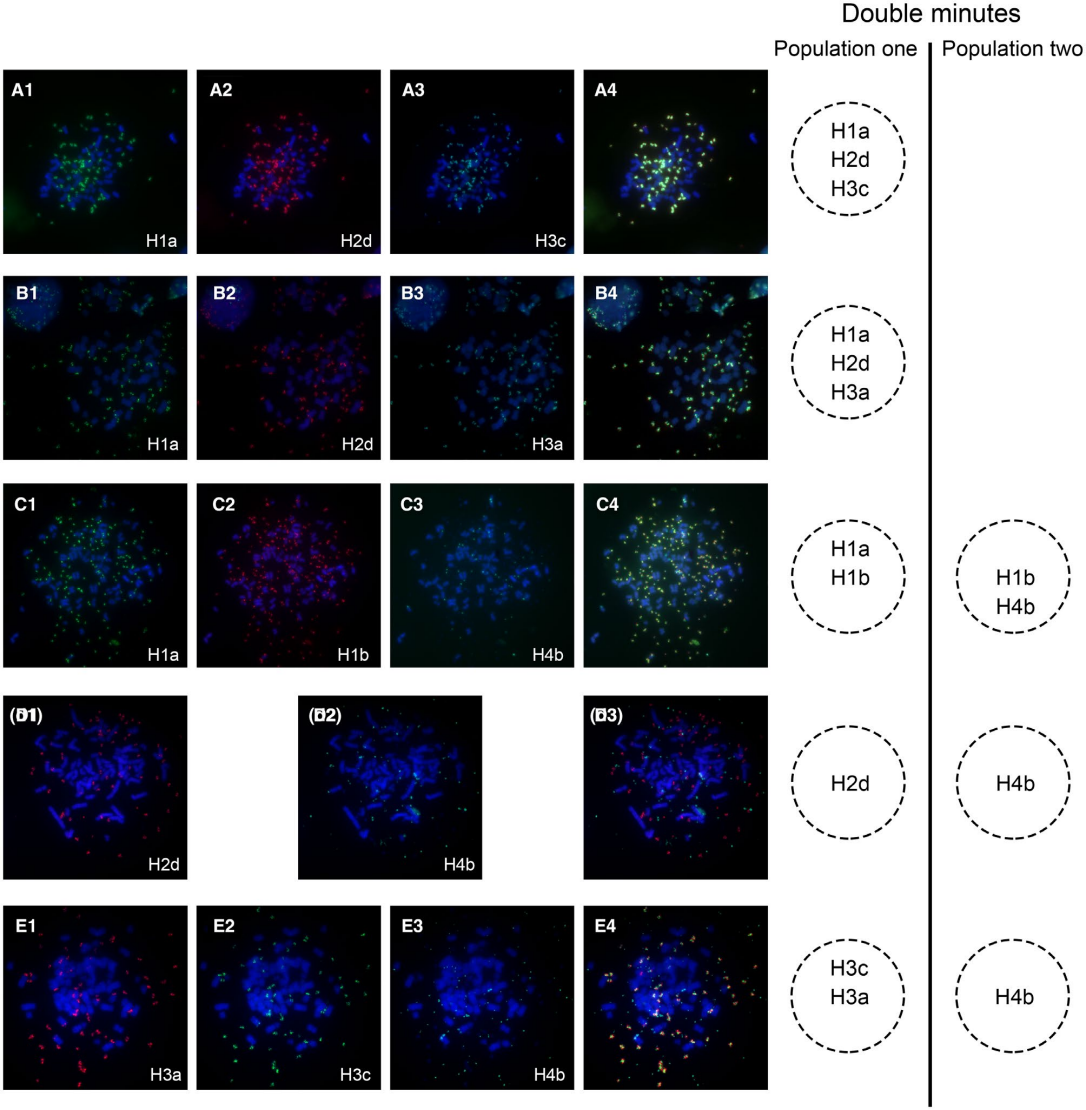
The heterogeneous composition of two subpopulations of DMs determined by M‐FISH analysis. (A) Co‐hybridization of RP11‐192N11 (green) (A1)‐H1a, RP11‐88J18 (red) (A2)‐H2d and RP11‐90G11 (light blue) (A3)‐H3c showed overlapping signals (A4) on the same DMs. (B) RP11‐192N11 (green) (B1)‐H1a, RP11‐88J18 (red) (B2)‐H2d and RP11‐691H24 (light blue) (B3)‐H3a showed overlapping signals (B4) in the same DMs. (C) Co‐localization of RP11‐192N11 (green) (C1)‐H1a and RP11‐585L3 (red) (C2)‐H1b; overlapping hybridization of RP11‐585L3 (red) (C2)‐H1b and RP11‐78A18 (light blue) (C3)‐H4b was shown in the overlay image (C4). (D) Hybridization of RP11‐88J18 (red) (D1)‐H2d and RP11‐78A18 (green) (D2)‐H4b disclosed that two BACs located on different populations of DMs (D3). (E) Hybridization of RP11‐691H24 (red) (E1)‐H3a and RP11‐90G11 (green) (E2)‐H3c demonstrated their co‐localization on the same DMs, but no overlapping signals with RP11‐78A18 (light blue) (E3)‐H4b was shown in the overlay image (E4)

Since DMs in the cells contained sequences from different chromosome fragments, we speculate that the way in which DMs arranged might be crucial to cell evolution. To this end, we chose six available BACs based on amplification level within these amplicons, as representative FISH probes to localize the sub‐amplicons in the cells. The amplified regions on DMs were verified by M‐FISH analyses, and co‐localization of the four amplified DNA fragments was shown in Figure 2. The results of M‐FISH confirmed the co‐existence of two populations of DMs in the same cell, which were defined as DM Population one, those containing amplicons H1, H2 and H3 merely from chromosome 8, and DM Population two which contained amplicons H1b and H4, derived from both chromosomes (8 and 10). Detailed observation revealed that the H1b signals, which appeared in all DMs, were further classified into two groups: group one co‐localized with the green signal representing Amplicon H1a, and group two co‐localized with the light blue signals representing Amplicon H4b (Figure 2C). Since the amplification level of these four amplicons (H1b, H2a, H2c, and H3b) was consistent, we considered that H2a, H2c and H3b also appeared in all DMs. This compositional discrepancy implicated the heterogeneity of DMs in NCI‐H716 cells. Meanwhile, we performed a DM counting on the karyotypes of M‐FISH assays. We found that the average number of two DM populations was 101 (Population one) and 107 (Population two), respectively (Figure S2). Combining with microarray data, we deduced that two populations of DMs in each accounted for around 50% in NCI‐H716 cells.

**Figure 3:**
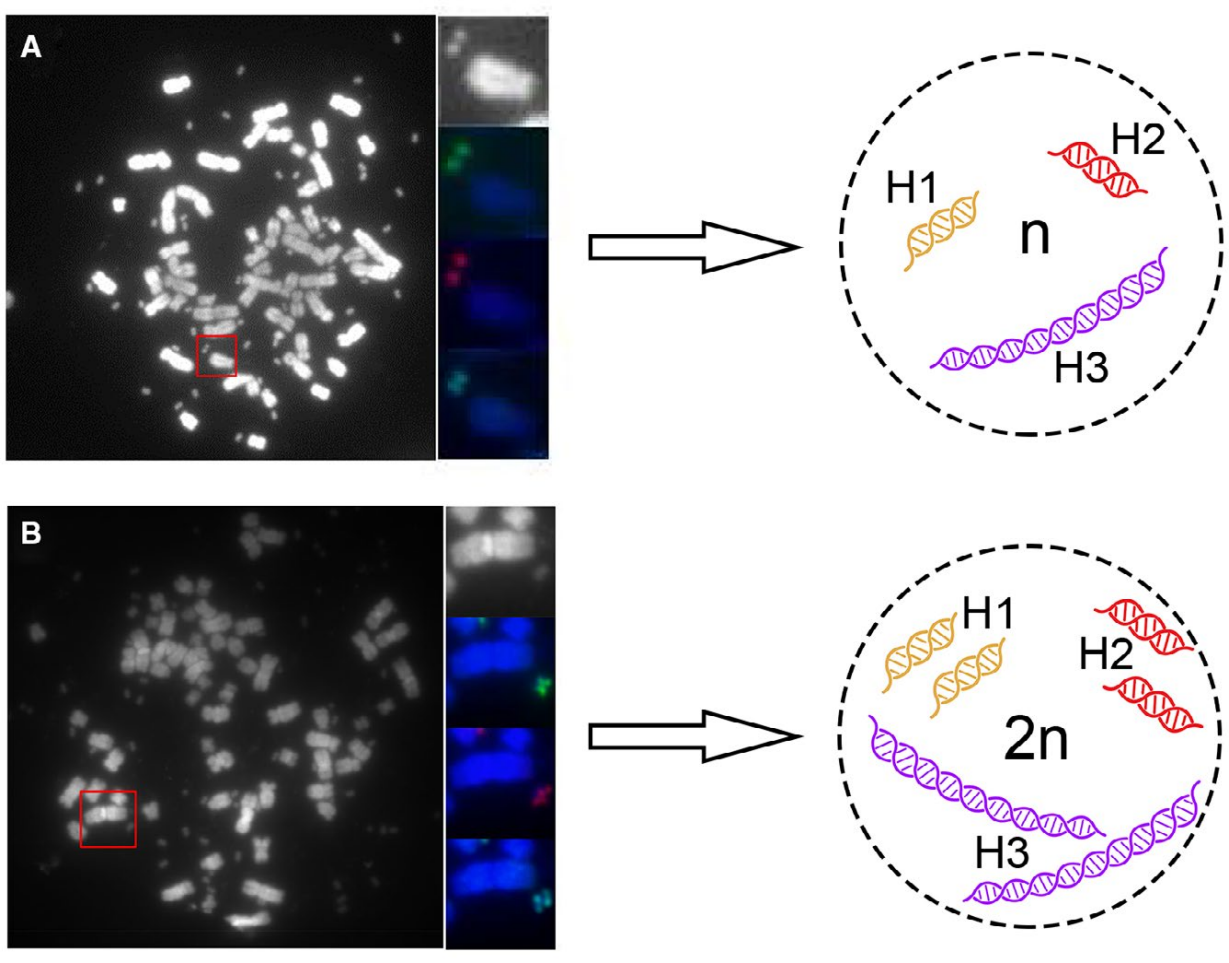
Two different molecular structures in Population one of DMs. Hybridization of RP11‐192N11 (green)‐H1a, RP11‐88J18 (red)‐H2d and RP11‐90G11 (light blue)‐H3c. (A) Uni‐fluorescence signal for each BAC probe was detected in one single DMs. (B) Two fluorescence signals were detected in one single DMs, which showed a structure similar to ‘diploid’

Furthermore, we observed duplicated signals of H1a, H2d and H3c located within one individual DMs from Population one, indicating a more complex molecular structure of DMs similar to a ‘diploid’ structure (Figure 3). However, these characteristic structures were not observed in DM Population two. We also analysed more than 200 metaphases and found that the fluorescence signals for each probe were specifically hybridized to DMs and no signal was detected in HSRs.

To investigate the heterogeneity of DMs in NCI‐H716 cells, we performed whole genome sequencing analysis for the sub‐amplicons (H1b, H2a, H2c and H3b) and identified a total number of 3,120,201 single nucleotide polymorphisms (SNPs), which including 1,689,436 heterozygote SNPs and 1,430,765 homozygote SNPs. We selected a total of 34 heterozygote SNPs in amplified regions from chromosome 8 for the study (Table S2) and found all of them were present in homozygosis in both subpopulations of DMs by alignment of M‐FISH and array‐CGH data. We detected 10 copies vs. over 1500 copies of 34 SNPs in heterozygote. Junction IV were located in both Population one and Population two, but chromosomes. These results suggested that two subpopulations of DMs might derive from the same chromosome 8, further confirming the nonhomogeneous configuration of DMs in NCI‐H716 cells. This has provided a novel frame on which we could further explore the impetus to drive DMs formation.

### 3.3 Fine sequential characterization discovered the junctions in DMs

**Figure 4:**
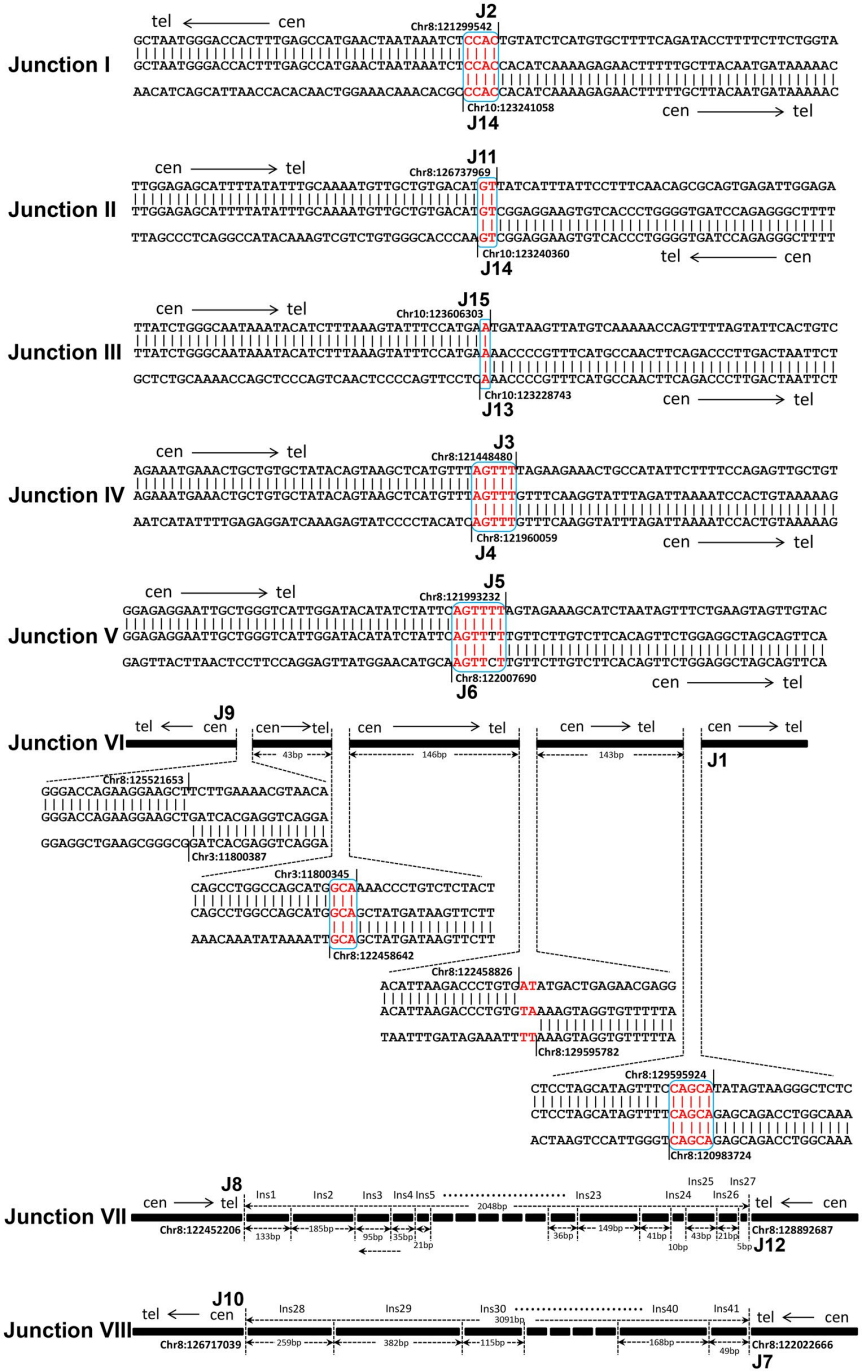
The sequence characterization of Junctions by alignment with human reference genome (GRCh37/hg19). Small insertions were shown in red. Microhomologies were shown in boxed red letters. Black vertical lines depicted the boundary of each amplicon. The size of each insertion in Junction VII and Junction VIII was indicated without scale. The complete sequences of eight junctions were listed in Table S3

Because of heterogeneity of DMs, we hypothesized that break‐rejoin model might be a possible mechanism to induce the occurrence of DMs. To elucidate the mechanism, we designed a NimbleGen capture array to detect the sequence of DMs. First, DMs DNA was enriched with a NimbleGen capture array and sequenced with PacBio RS system with average read length of 1100 bp on the basis of circular consensus sequencing. The sequencing reads were then aligned to the human genome to obtain the breakpoint sequences. On the basis of the CGH microarray results, 15 breakpoints within the above four major amplicons were identified and named J1‐J15 in sequential order (Figure 1). Among these breakpoints, the sequences of 11 breakpoints were completely obtained including J1, J2, J3, J4, J5, J6, J9, J11, J13, J14, and J15. Six types of junction (Junction I to Junction VI) were then predicted (Figure 4), and the sequence around the joining site was shown in Table S3. To fill in the gaps existed in the target regions generated by capture sequencing, whole genome sequencing with average read length of 150 bp was performed with an Illumina HiSeq X Ten Sequencing System, which produced 113 Gb raw Paired‐End sequencing data obtained with 30X sequencing depth. Finally, most of sequence gaps on target regions were covered, but sequences in the rearrangement breakpoints J7, J8, J10, and J12 were still not available.

To acquire the entire sequence map of DMs, junction sequences must be fully ascertained. Therefore, we cloned the rearrangement breakpoints J7, J8, J10, and J12 of DMs by chromosome walking technique starting from the boundary sequence obtained through CGH microarray and high‐throughput sequencing. Then, the rejoining patterns were manifested by alignment of the cloned sequence to the human reference genome (Figure 4). As shown in Figure 4, Junction VII and Junction VIII, in sharp contrast with other Junctions, had more complicated sequence with multiple DNA fragment insertions running 5 bp to 679 bp in length (Table S4). Moreover, most of the insertion fragments (18 out of 20) in Junction VII originated from non‐amplified regions of chromosome 8 without considering uncertain results, and 2 insertion fragments were from the amplified regions which were 110 bp and 5 million bp away from the insertion position. On the contrary, all insertion fragments originated from the amplified regions of chromosome 8 or 10 except for uncertain data in Junction VIII (Table S4).

### 3.4 The cyclic molecular structures of two distinct DM populations in NCI‐H716 cells

**Figure 5:**
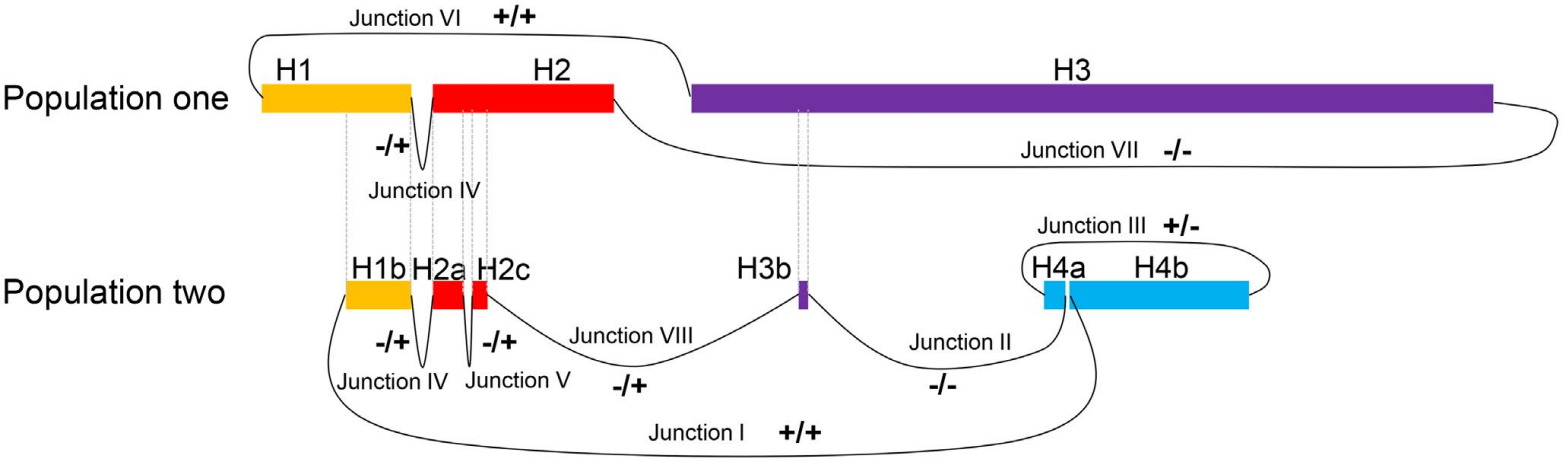
The rejoining model of rearranged fragments in two subpopulations of DMs. All fragments of DMs were shown from left (‘+’‐head) to right (‘−’‐tail). All rejoining models were listed and marked as −/+ (tail‐to‐head), +/− (head‐to‐tail), +/+ (head‐to‐head) and −/− (tail‐to‐tail)

**Figure 6:**
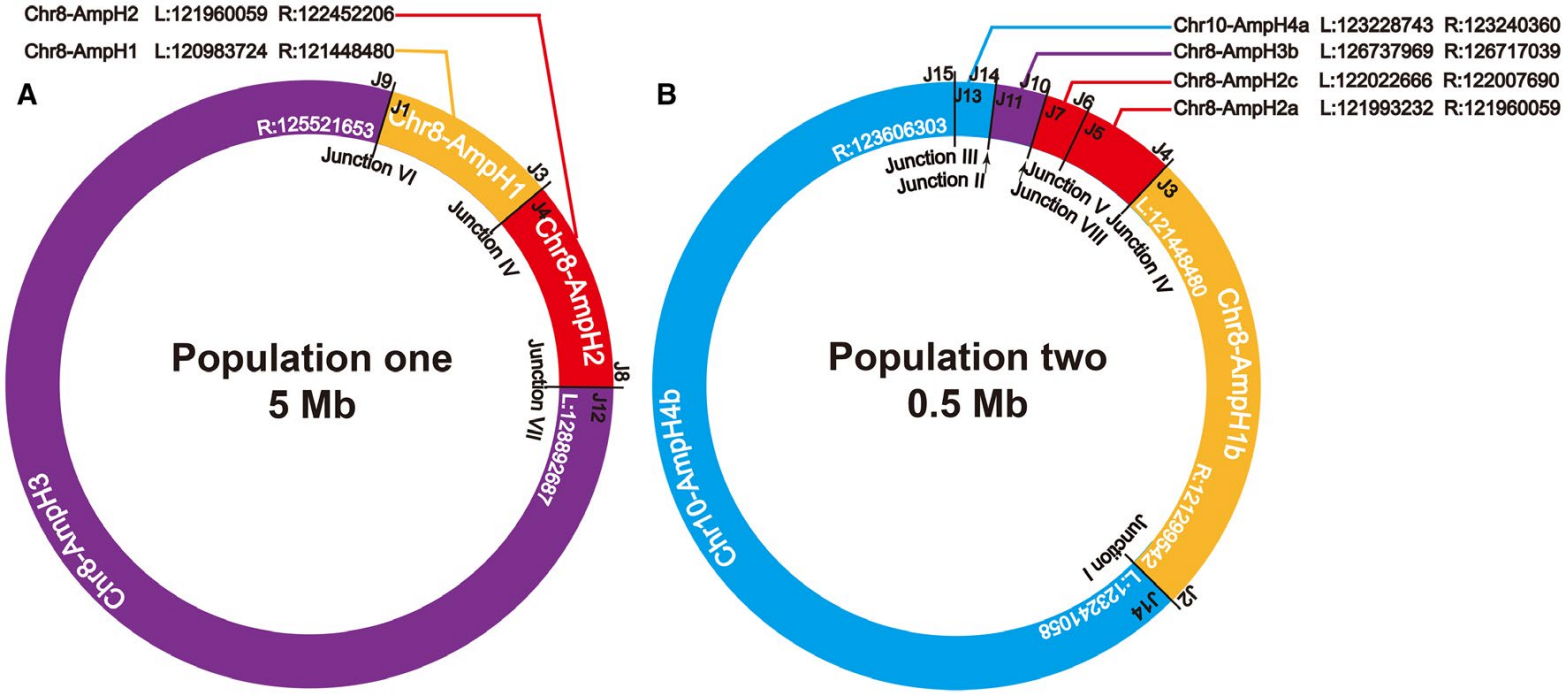
The molecular structure model of two subpopulations of DMs. (A) Circular model of DM Population one, which was around 5 Mb in length, was summarized by sequence alignment and M‐FISH. Corresponding positions of rearrangement breakpoints of amplicon H1, H2 and H3 in human genome were indicated as L and R. (B) Molecular structure model of DMs Population two showed the circularized compositions of amplicons H1b, H2a, H2c, H3b, H4a and H4b. The chromosome origins and the name of the Junctions were listed inside the circles

The regions of each amplicon in DMs were validated through high‐density array CGH. The amplified regions on DMs were verified by M‐FISH analyses, and the results confirmed two populations of DMs in NCI‐H716 cells. Precise boundaries and junction sequences of each amplicon were identified by high‐throughput sequencing and chromosome walking analysis. Based on our research data, we reconstructed the molecular structure of DMs (Figure 5). In detail, the complete Amplicons H1, H2 and H3 were rejoined to form DM Population one (Figure 6A), and the Amplicons H1b, H2a, H2c, H3b, H4a, and H4b formed DM Population two (Figure 6B). Therefore, the Amplicons H1b, H2a, H2c and H3b were shared fragments of both DM populations. As a result, the copy numbers of these four amplicons were much higher than those of the others. These results were consist with the microarray results indicating that these four amplification regions (Amplicon H1b, H2a, H2c and H3b) had higher copy numbers (log_2_ ratios ≈ 7) than those in the other amplification regions (Amplicon H1a, H2b, H2d, H3a, H3c, H4a and H4b, log_2_ ratios ≈ 6).

## 4 DISCUSSION

Previous studies have reported the existence of endogenous DMs carrying fragments from chromosome 8 and 10 in NCI‐H716 cell line,^22^, ^23^ providing us with a preliminary model to configure the underlying mechanism of DM formation. In this study, we analysed copy number variation and structure variation of DMs in human colorectal cancer NCI‐H716 cells using a specifically designed high‐resolution microarray. The high probe density is required for the accurate detection of subtle variations in the amplification regions. Copy number variation of different amplifications provides a solid basis for BAC selection. For instance, the amplicon H1 had two amplification regions with different copy numbers. The sub‐amplicon H1b with higher copy number existed both in DMs Population one and Population two. Finally, we identified that the amplicons H1b, H2a, H2c and H3b were shared fragments of both DM populations. Since the BAC selection in different amplification regions, our result was different from previous studies that the two populations of DMs originated from completely different chromosomal segments. However, as confounding marker of chromosome instability determining cell fate, DMs are more complex than current understanding. Much detailed depiction about the biological feature and utility of DMs is still needed to be clarified.

Our study employed the high‐throughput sequencing technique, providing an unprecedented platform to detect DMs in cancer cells.^16^, ^17^, ^18^, ^24^ However, this technique has limitations in current study. Like, the sequence data generated by PacBio RS and Illumina HiSeq X Ten Sequencing system only presented partial junction sequences of Junction VII and Junction VIII. These incomplete information led to misinterpretation that the rearrangement breakpoints were connected to chromosomal segments (ie insertion fragments in junction VII), and thus, the highly amplified regions were inserted into chromosomes to form HSR, which was inconsistent with the M‐FISH‐based results. Therefore, in order to obtain the complete junction sequences, we replenished the sequencing data with chromosome walking and long‐distance PCR, which successfully filled in the gap sequence left by high‐throughput sequencing. Our results have the important implication that comprehensive application of multiple complementary techniques may be more powerful for studying the structure of DMs. Some recent studies tried to establish the framework for detecting DMs on the basis of high‐throughput sequencing data. The high‐throughput sequencing results contained all DNA sequences of chromosome and DMs, and thus the heterogeneity of DMs should be received adequate attention at the same time. Based on FISH method, the wide exist of subpopulations of DMs had been demonstrated in cancer cells.^10^ Understanding the heterogeneity of DMs will enhance our understanding of cancer evolution and potential treatment strategies.^25^ Thus, it is very important for us to examine the number of subpopulations and its composition of high‐amplification regions before constructing the molecular structure. M‐FISH analysis is a key technique, which is a fundament of molecular cytogenetics for molecular biology.

In this study, we aimed to reveal the mechanisms of DMs formation by deciphering the junction events. As a result, we found three ‘junctions’ (Figure 6A) in DM Population one, and six ‘junctions’ (Figure 6B) in DM Population two with Junction IV shared by both populations. In total, nine recombination sequences (Junction V has four recombination breakpoints) were found to mediate these Junctions, excluding Junction VII and Junction VIII. Of the nine junctions forming Junction I to VI, eight were recombinated by MMEJ and one by blunt end joining, which are two major forms of NHEJ pathway.^26^, ^27^ It was consistent with the DMs described in human ovarian cancer cell line UACC‐1598.^28^ NHEJ, as a simple recombination‐based mechanism, can explain some nonrecurring rearrangement.^26^, ^29^ However, Junction VII and VIII showed more complexity compared with Junction I to VI, with most insertions inside junction sequences originating from the amplicons or adjacent chromosome regions, which cannot be simply explained by NHEJ mechanism. This characteristic has provided evidence of DNA replication‐based mechanisms—the FoSTeS and the MMBIR model, which have been proposed to explain the formation of complicated rearrangement in human genome. During DNA replication, the lagging strand disengages from the original template, transfers and then anneals, through microhomology at the 3' end, to another replication fork with physical proximity and restarts the DNA synthesis, thus leading to multiple distinct regions with complex rearrangements.^30^, ^31^ The rejoining pattern of these junction sequences suggested that both NHEJ/MMEJ mechanisms and FoSTeS/MMBIR mechanisms might be involved in the formation of DMs in NCI‐H716 cells.

Till now, several different models have been proposed for the formation of DMs.^32^, ^33^, ^34^, ^35^, ^36^ Among them, the chromothripsis generation model as a one‐off genomic catastrophe has been proposed on the origin of complex DMs.^37^, ^38^, ^39^ Thorough understanding of this theory disclosed six criteria prerequisite for chromothripsis,^40^ among which at least two criteria were needed to invoke chromothripsis. One criterion was ‘clustering of breakpoints’, and 5 to10 breakpoints should be located within 50 kb genomic intervals for chromothripsis to occur. In NCI‐H716 cell line, Junction VII and VIII presented this type of breakpoints clustering, which included 28 and 15 breakpoints, respectively (Table S3). The other criterion is ‘randomness of DNA segment order and fragment joins’, meaning that chromosome fragments are randomly joined and distributed with uniform intervals. Our results meet these requirements by showing locally uniform joining and ordering of fragments in the eight junctions observed in DMs, with the numbers of tail‐to‐head (−/+), head‐to‐tail (+/−), head‐to‐head (+/+) and tail‐to‐tail (‐/‐) rearrangements being 3, 1, 2 and 2, respectively (Figure 5). Therefore, the sequential features of DMs in NCI‐H716 cells partially reflected the feasibility of chromothripsis model.

**Figure 7:**
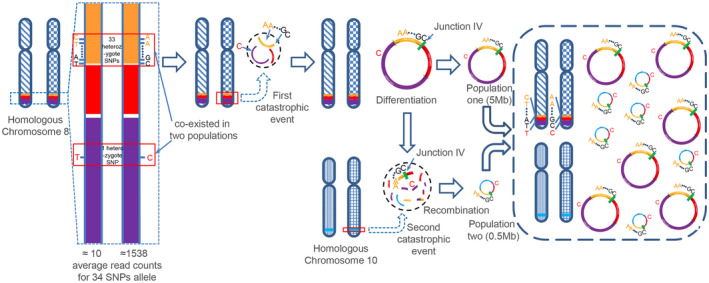
Process of multi‐step evolution drives DMs formation in NCI‐H716 cancer. The yellow strip, red strip, purple strip and blue strip represented each corresponding amplification region—H1, H2, H3 and H4. Green bar represented Junction IV, which does not exist on chromosomes, shared by both populations

In NCI‐H716 cells, DM Population one might have participated in the formation of DM Population two. It can be speculated from the following evidence. First, DM Population one included three integral amplicons (H1, H2, and H3); in contrast, DM Population two only contained partial sequences of these amplicons (H1b, H2a, H2c and H3b). Second, more powerful evidence was from the formation of Junction IV (green bar), which does not exist on chromosomes, shared by both populations (Figure 7). Finally, 34 SNPs of chromosome 8 we identified were present in heterozygous with 10 copies vs. 1,500 copies (Table S2). These SNPs were present in homozygosis in two populations of DMs. It suggested that these amplicons co‐existed in two populations derived from the same chromosome 8. Thus, we speculate that subpopulations of DMs might be formed by rearrangements in a stepwise manner. That is, in the early stage of DM formation, a first catastrophic event, chromothripsis ^37^, ^38^, ^41^ for instance, occurred in chromosome 8 and aroused fragmentation and replicative repair within the chromosome. Most chromosomal fragments are eliminated extracellularly during the cell cycle, while the retained fragments may be further recombined and circularized to form DM Population one. The generation of Population two might arise from a second catastrophic event, which integrated some specific sequences from Population one with the fragments of chromosome 10. Through replication, unequal segregation of DMs and selection for growth advantage, the cancer cells carried numerous specific DMs. Thus, we speculate that subpopulations of DMs might be formed by rearrangements in a stepwise manner, rather than with independent evolution or one‐off genomic catastrophe.

In summary, we re‐identified two populations of DMs in human colorectal adenocarcinoma cell line NCI‐H716, confirming their heterogeneity in cancer cells and managed to construct their molecular structure, which were not investigated before. Based on our analysis, we propose that both NHEJ/MMEJ and FoSTeS/MMBIR pathways may mediate the rearrangements in DMs, and the complex structure of DMs in NCI‐H716 cell line may be generated by multi‐step evolutionary process involving various mechanisms. Further anatomy on DMs will enhance our understanding the biological significance of extrachromosomal chromatins in cancer.

## CONFLICT OF INTEREST

The authors declare that they have no competing interests.

## AUTHOR CONTRIBUTIONS

**Xueyuan Jia:** Conceptualization (equal); Methodology (equal); Software (lead); Writing – original draft (lead); Writing – review and editing (lead). **Rongwei Guan:** Data curation (lead); Software (equal). **Xiaobo Cui:** Data curation (equal); Software (equal); Writing – review and editing (equal). **Jing Zhu:** Methodology (equal); Software (equal); Validation (equal). **Peng Liu:** Methodology (equal); Validation (equal). **Ling Zhang:** Visualization (lead). **Dong Wang:** Methodology (equal); Software (equal). **Yang Zhang:** Conceptualization (equal); Methodology (equal). **Kexian Dong:** Methodology (equal); Software (equal). **Jie Wu:** Validation (equal); Visualization (equal). **Wei Ji:** Methodology (equal); Validation (equal). **Guohua Ji:** Resources (equal). **Jing Bai:** Project administration (equal); Supervision (equal). **Jingcui Yu:** Resources (equal); Supervision (equal). **Yang Yu:** Conceptualization (equal); Supervision (equal). **Wenjing Sun:** Data curation (equal); Resources (lead); Validation (lead); Writing – review and editing (equal). **Feng Zhang:** Conceptualization (lead); Funding acquisition (equal); Methodology (lead); Supervision (equal). **Songbin Fu:** Funding acquisition (lead); Project administration (lead); Supervision (lead); Writing – review and editing (equal).

## Supporting information

Supplementary Material

## Data Availability

The data from this study have been submitted to the NCBI Sequence Read Archive (SRA; https://www.ncbi.nlm.nih.gov/sra) under accession number SRP127522 and Gene Expression Omnibus (GEO; https://www.ncbi.nlm.nih.gov/geo/) under accession number GSE110071.
