# Supplementary material for: Molecular structure and evolution mechanism of two populations of double minutes in human colorectal cancer cells
Source: J Cell Mol Med. 2020 Oct 30;24(24):14205–16. doi: 10.1111/jcmm.16035 (PMC7754069; doi:10.1111/jcmm.16035)
Supplement: Supplementary file 1 — Supplementary Material [file JCMM-24-14205-s001.pdf]

Supplementary Table S1: BACs or Fosmids used for FISH analysis

BACs	Chromosome band	Chromosome position Start	end	Amplicon position
RP11-192N11	8q24.12	chr8:121,053,046-121,242,086		AmpH1a
RP11-585L3	8q24.12	chr8:121,329,967-121,490,786		AmpH1b
RP11-88J18	8q24.12	chr8:122,190,052-122,368,700		AmpH2d
RP11-691H24	8q24.13	chr8:125,548,470-125,767,407		AmpH3a
RP11-90G11	8q24.21	chr8:127,574,946-127,751,550		AmpH3c
RP11-78A18	10q26.13	chr10:123,418,495-123,577,576		AmpH4b

Supplementary Table S2: SNP allele in chromosome and four amplicons (H1b, H2a, H2c and H3b)

Chr	CytoBand	Position	Allele 1 /Allele 2 in chromosome	Allele in DMs	Number of reads for Allele 1	Number of reads for Allele 2	Amplified regions
8	8q24.12	121302877	C/A	A	15	1623	AmpH1b
8	8q24.12	121303988	T/A	A	6	571	AmpH1b
8	8q24.12	121306354	A/G	G	4	996	AmpH1b
8	8q24.12	121307025	C/T	T	4	1323	AmpH1b
8	8q24.12	121308413	T/C	C	14	1363	AmpH1b
8	8q24.12	121309679	A/T	T	3	677	AmpH1b
8	8q24.12	121309862	A/T	T	6	974	AmpH1b
8	8q24.12	121310102	C/T	T	17	1269	AmpH1b
8	8q24.12	121310561	G/A	A	5	819	AmpH1b
8	8q24.12	121313629	T/C	C	11	1867	AmpH1b
8	8q24.12	121315402	G/A	A	25	2015	AmpH1b
8	8q24.12	121317359	A/G	G	11	1431	AmpH1b
8	8q24.12	121318493	T/A	A	10	1318	AmpH1b
8	8q24.12	121320402	A/G	G	7	1324	AmpH1b
8	8q24.12	121320951	A/G	G	12	1917	AmpH1b
8	8q24.12	121321313	G/A	A	6	1420	AmpH1b
8	8q24.12	121322653	T/C	C	17	1987	AmpH1b
8	8q24.12	121324191	C/T	T	4	916	AmpH1b
8	8q24.12	121325618	A/C	C	28	2146	AmpH1b
8	8q24.12	121325670	A/G	G	16	2368	AmpH1b
8	8q24.12	121326427	G/T	T	11	1885	AmpH1b
8	8q24.12	121326616	T/G	G	11	1558	AmpH1b
8	8q24.12	121328547	C/T	T	11	1638	AmpH1b
8	8q24.12	121350362	G/A	A	11	1660	AmpH1b
8	8q24.12	121384119	T/C	C	18	2145	AmpH1b
8	8q24.12	121386569	A/G	G	3	2087	AmpH1b
8	8q24.12	121391011	T/G	G	3	2037	AmpH1b
8	8q24.12	121394026	A/G	G	13	1955	AmpH1b
8	8q24.12	121401398	T/A	A	3	1431	AmpH1b
8	8q24.12	121401888	T/C	C	3	1459	AmpH1b
8	8q24.12	121408195	T/C	C	3	1316	AmpH1b
8	8q24.12	121433224	A/G	G	3	1041	AmpH1b
8	8q24.12	121447555	T/C	C	4	2000	AmpH1b
8	8q24.13	126728809	T/C	C	16	1754	AmpH3b

Supplementary Table S3: Joining sequences in each junction

Name	Break-points	Sequencing of the breakpoint regions
Junction I	J2/J14	AGAAATTAAGATAAAGTATTTATTCATTTATAAGTCTATGAACAAATAGAATTCGACTTC<br>TACAAATTGAATTTGCATTGAGATATTCTATTAATAGGTCTATTATAAAGTATTTTGTCAC<br>ATGAGAAGCCTTTTTTGAATTTTTTTCCTTGTTTGGCTTTTTGGCTTTATTAACACTTAA<br>AGTAGAGGGAAGTCTTCCTTCATATAACTTTTAAAAATTTGAAGGTTTATAAATTTTAA<br>ATCGATTTTTAATGAACCTCACTATTTGAGTTCTTCTGGTAAGGAAATAAAGCTTAACC<br>TTCACCTAAGACTTACAAAGGATTCTTTGGAATGAGAATTACAAGTTAATGCCTAAAC<br>CATGGGCTAAAATGACAATGTTTCTTTTAACACTCATGAAATCTCAAACGCTTTTCATC<br>TCCCAAACAATTTTATTTTTTACTATTTTGCAATACACTCTCCATAACCAAGAAAATAAA<br>CATGCCAAGAGGAATTTGGTGAGTAAACAATGTTAAGTCCTAAGAGCTGCTAATGGGA<br>CCACTTTGAGCCATGAACTAATAAATCTCCACCACATCAAAAGAGAACTTTTTGCTTA<br>CAATGATAAAAACGAAATTTTGTCCTAAATGGAACCGTTTTTCTTGAGCATATGGTAAT<br>GATTTTCAGAAGGAAAGAAACTTCGATTTTTATATCCACCAGACAATATGGCATTCTGC<br>TTTTTTATTTTAGGGTACTAGATACAAATTTCAGTATCAACGGACCCACTCTACATGTAA<br>ATATATAAATAAATCACTTTGAGCATAGACATGAGATCATTAAAATAGAACTTTAGTGCT<br>TTTCCCAGTTTCTTTCTTTTTTATTTTTATTTTTTTTTTTTGAGATGGAGTCTCGCTCTGT<br>CGCCAAGGCTGGAATGCAATGGCATGATCTCGGCTCACTGCAACCTCCGCCTCCCCGG<br>TTCAAGTGACTCTCCTGCCTCAGCCTCCCGGAGTAGCTGGGATTACAGGCGCACACC<br>AACACGCCCGGCTAATTTTTTGTATCTTTAGTAGAGACAGGGTTTCACCATGTTGGCCA<br>GGCTGGTCTGGAACTCCTGACCTCATGATCCGCCTGCCTCGGCCTCCCAAAGTGCTGG<br>GATTACAGGCATGAGCCACTGCGCCTGACCAACTTTTCCCAGTTTCTCAATGAAGCCA<br>TAAACTTTCAGATCTGATAGGAAAAAAACAGGGATATCAGTAGATT
Junction II	J11/J14	CAGCTGGCTGGAGTTTAATCTGAAGTTCTGGGGGCAGACCGCAATTGCTGCCACAGA<br>GCCCCTCCTGGCAGGTGCAGCCCACAGGAAGACCAGCACCCTGGCCACAGAGACAA<br>AAGGTCTTATTGACTTGCAGTAAAAATGTCCTTATAACCTGTCCAGAACAAAAGGGGA<br>GGATAAGGTTCAACCAGACACCGAATTCAGGGAGACAGCATCAGTCAGAGCTTTACC<br>ACTGCCAGCAATTTTGAAGCTGCCCATATTTCTGCTCAGGCAAGATGTTTTCTTGCTCT<br>GAAACCTTTTAGCAGATGCAAGGTATTGCTCCGGGTGTATCCCAGAACACAAGTCTTT<br>GCTAAAGTGGGTAGACAGCCACTTTCCTTCAGAGCCAGGGACTGCCCTGGGAGTAAA<br>ACCATTTGGAGAGCATTTTATATTTGCAAAATGTTGCTGTGACATGTCGGAGGAAGTG<br>TCACCCTGGGGTGATCCAGAGGGCTTTTTGCAGCACCAGCACTGGATGCCTTGGGGA<br>GCAGAGGACACTTTCTGAATCTCGAACCTTCAAAACTTTGAAACTTAACCGATATTTT<br>CCTGAAGTTTCCTTTAACAGTGCATTATGGATTTAACATACATGAGTATGTCTAAGCCTT<br>TTTTGAACCTGTTTACATTTTCAACTCGGAGAGCTTCTGAGAGTAATCAGTTACATCTG<br>TTAAATTACCCACTGAATGAAGAAGTATTCCCCTTCAATTATCCCCGAAACTACAATAG<br>TAAAATGTAATTCAGAAAATAAATATTCATAGTCTCAACTACTTAAAGGGGGTCTGGGG<br>ACCCACAACATGTTACCGGTTTTCATTTTGCTGGGGCTCGCATTTGAAGCAAGGGCTC<br>TAGTGAGGAGGTGGTGTAGA
Junction III	J15/J13	CAACCAGTACAGATTTCTTTGTTCCTTCTCCATTCCCGCTGCTTCATTTGACTAGCCTG<br>CCTAAATAAATAAATAAATAAATAAATAATAAAATGTTATCTGGGCAATAAATACATCTT<br>TAAAGTATTTCCATGAAAACCCCGTTTCATGCCAACTTCAGACCCTTGACTAATTCTCT<br>GAAGAAATTCTGGAACCATCCATGTTCCCCCACACCCCGCTCCCCCACTACCCTTCTT<br>TCTTACTGGCTGCTGTGCTCTTCTGCAGCTGTAGGGATGATCTGGACTCCCCTCCTAAG<br>CTGCTTTTGACGCATGTTGGAGATCTTAAATAACCAGAAGGCCCTACGTCGTGGGGCC<br>ACTAGAAGAATTCTGAATGTTTTTATTGTTCTTAAAAGCTGTGAAACCCTGGTCTACGA<br>CAGTGCATAGGCATGCACACACACACACAACCACAAATACCTGCCTGGCCCCTGGCC<br>CAGGAGCCCCTTCTCAGGTTCTCCCTGGGTCTACAGGTGCCATGTAAGTCAGAGCAG<br>CCCAACAATGCGGGCTCCAGCCTCGCCTGCTCCCTCCCTCCTGCCCAGTCTTTAAAAA<br>GAGTCTACGCTTTCACCCTGGGGATCTGCCCAAGGCTTTGCTTCCCCAAAGAGGCTAT<br>GAACACCCAGGTTTCCACTATTAGGGGTCCCCCCTTCATCTGCATATCTCCTGCTCTTG<br>ACCACATTCACCTGCTTTCAGATCAGAAAAATAAGGGCTTAGTGCTTGATTTGCTTTC<br>AGCTTCATTTAGAAGACAAGGGAAAAACTGAAAGCAGATCCAAGCATTTTTATTACCA<br>GTCTCCAGTACCTGTACAC

Junction IV	J3/J4	GTGTATATGTACCACATTTTCTTTATCCAGTCTACCACTGATGGGCACTTAGGTTGATGA<br>CGTATCTTTGCTATTATGAACAGTGCTAAAATGAACATATCTGCATGCTTTAAGTTCTTT<br>CAGAAATCACCAAATTGCTTTCCAGAATGACTGAACTAATTTGCACTCCCACCAGCAG<br>TGTAAAAGCATGCCCTTTTCTCCTCAGCCTTGCCAATGTCTATTATTTTTTGACCTTTTA<br>ATGATAGCCATTCTGACTGGTGTGAGATGGTATCTCATTGTGGTTTTGACTTACACTTC<br>TCTAATCATTAGCGATATGGGCATCTTTTCATTTGCTTGTTGACTGTATGTAAGTCTTCT<br>TTTGAAGTGTCTCTTTATGTATGTCCTTTGCCCACTTTTTTATAGGGTTGTTTTTTGCTT<br>GTAAATTTGTTTAAGTTCCTTACAAAGAAATTCTTGTTAATCAATCTTATGCTCTAGTTG<br>AAAAGCCAGACAATAAACAAACAAATAAATGAATATGCAATATGCCAGGAGCTGATG<br>AACATGGGAAGATAAAGAGTAATAGGGTGAAGCGATTAATTGGTACAGGTTTTTCATG<br>AACTTAATTTTCATTTCTCTGGGATAACGTCCAAGAGAAATGAAACTGCTGTGCTATAC<br>AGTAAGCTCATGTTTAGTTTGTTTCAAGGTATTTAGATTAAAATCCACTGTAAAAAGTG<br>CATTTTTATATCACACACAGAGCATACGCAGTGCTCACACACGCACATACACATAACTG<br>AACCAAGGATTTACTTAAAAATACATATCCTTACTGTATTAATTCGTTCTTACACTGCTA<br>ACAAAGACATACCTGAGACTGGGTAATTTATAAAGAAAAAGAGGTTTCATGAATTCAC<br>AGTTCCACGTGGCTGGGGAGGCCTCACAATCATGGCGCAAGGCGAAGGAGGAGCAA<br>AGTCATGTCTTATGTGGCGGTAGGTAAGAGTGTGTGCAGGGGAACTGCCCTTTATAAA<br>AACATCAGATCTCATGAGACTTATTCACTCTCACGAGAACAGCATGGGAAAAACCCGC<br>CTCCTGATTCAATTACCTCCTGCTAGGTCCCTCCCATGATGCATAGGAATTATGGGAGC<br>TACAATAAAAGGTGAGATTTGTGTGGGGACACAGCCAAACCATATCACGAGGATTTAC<br>TTAAAAATACTTATTCTGGGCTGGGCACAGTGGCTCATGCCTGTAATCCCAGCACTCTG<br>GGAGGCCGAGGTGGGCAGATCACCTGAGGTCAGAAGTTCAAGACCAGCCTGACC
Junction V	J5/J6	GAAAAAATGAAAAAAGGTAGCCTTGTGCATATGGAGAATGGGAATGCTCTGTGTGGA<br>TGGTTGCATTCCAGTCATATTTCCAAGTTTACCCTAAAGCCACAGATTTGGTTGTATGG<br>AATTCAGAAGTTCTAATTAGAAATATGAAGAAGGATATGGACTTCCTTGTATATTTTTA<br>AATTGATATACAAACTACATTTGGTAAAGTGAACAAACCTTAAGTTTAAAACTTAATGA<br>ACTTTACAGAGTGAACACATTTGTGTAACCCCATCCAGATAAAAATAGAGAACACTGT<br>CAATAGTACAGAATCATCCCTGCCTCCTTCAGCCCTTCCTACCTGTGCCAAAGATAACC<br>ATATCCTGGCTTCTGTTACCCTAGGCTAGTTTTGTCTTTCTGAATTTTACATGAAAGGAA<br>TCATAGAATTTGTGTTTTTTGCTCAACATCATGTTTTTGAAATTCATTTATACCATTGCAT<br>AGAGCAGTTGTTTCTTCCTTTTATTGCAGTATTGTATCCCATTGTGTGAATATATTACCA<br>CAATGTTATTTTCCACTGTTTATGGACTTTTGGGTCAATTCTCATTTTTGGTTATTCACC<br>ATAATACTGCTTTAAGCATTCTTGAATATCTCTTTTGCACAAATCTGTACACTTATGCAT<br>TTCTTTTGGGAATGTATCAGGAGAGGAATTGCTGGGTCATTGGATACATATCTATTCAG<br>TTTTTGTTCTTGTCTTCACAGTTCTGGAGGCTAGCAGTTCAAAATCAAGTTGTCAGCA<br>GGGTTGTCCCTCCTGAGGACTTTCCCCTTGGCTTGCTGATGGCCACCTTCTCCCTATCC<br>TCACATAGTCTTTCCTCTATCCAGGCTCATCCCTGATGTCTCTTTGTGTGTCCAGATTTC<br>CTCCTCTCCTAAGGACACTAACCAGATTAGGTGGGGGTACCCTCTAATGGTCTCCATTT<br>AATTTTGTCACTTCTTTAATTGCCCTATCTCAAAATACAGTCACATTCTGATGTACTGGA<br>GGTTAAGGCTTTATCATATAAATTTTGGGAAACACATTCAGCCCACAACACTCTCCCTT<br>GGAGGTGGCAGATGTCATGCCTGCTCAAAATGCTTTGGTCAGAGCAAGTCACATGATA<br>ATGAATGGGGGCTGTATAATCCTCTGAGAGGGGAGGGAGGAAATTCACTTGAGGAAT<br>AATAGTGGCTTCTACTCAAGAATAGATTTGTCTATACTTTTGGCTACTCCATCTTATGCA<br>A
Junction VI	J9/J1	GCTGGGATTACAGGCATGTGCCAACACGCCGGCTAATTTTTGTATATTTAGTAGAGACG<br>AGGTTTCACCATGCTGGCCAGGCTGGTCTTGAACTCCTGCCCTTGAGTGATCCACCCG<br>CCTTGGCCTCCCAAAGTGCTGGCATTACAGGTGTGAGCCACCACGCCCGGCCTGAAG<br>ACTGCTTCTTAAGAAGTACATTTCTATTTCTAATTTTAAAATTTAGCATTTTCAAGAGAG<br>CAAAGGGGGTAAAATAGTCCTGCACATCAGGAAATATGATATAGAAAAATATAGCAAG<br>AGAGGCTTAAGTTAAACAGAGAAATTGTTTCCCCAAATTAAAGTGCTTAGAAGAGGT<br>AAGCACATTTAAGAAAATAATAAGCCACCTGTTATCCTGATTCAGTTGAACCTACCATT<br>TGTGAGAACAAGGGGGAGCACTGTGTTGAAGTGAAAAGACAAACAGAATGAACATT<br>CGTTGTATCCTCTGAATATTGTCATTATACTCCGGGACCAGAAGGAAGCT▲GATCACG<br>AGGTCAGGAGTTCAAGACCAGCCTGGCCAGCATGGCA▲GCTATGATAAGTTCTTTCA<br>CAGAGAGAACCATGGTGCCATGAATGCACCTGGTAAAAATGTGAATCAGCCATGGCT<br>TCTCTGAGGAAATGGAATTGACATCTGAGGAAGGTAAAAGGCAATGAGTCAGGTGGG<br>AGTGAAGAAGATTCCAGGCAGGTAGAACAGCATACATTAAGACCCTGTG▲TAAAAGT<br>AGGTGTTTTTAAAAAATCCTTTTTAGAGACCTCATTTACTTACACATTCACAAGGCTGG<br>ACTCCAAGGCCTTCAAAGAGCAAAAAGTACACTTAATTATTATTCAGAGTTTATATAGC<br>TCCTAGCATAGTTTTCAGCA▲GAGCAGACCTGGCAAAAGTCTGACCAGGTTTGTCCA<br>CAGACTTCAGTCTTTCTTCCTGTCATATGAGTTCAGTTAATGAAAACTCAGGGAAGGA<br>ACCAGAGGTAAATTGTTTTCTTCTTTGGCAGGTCCAGACTTAGGCAGGTAAGGAACTT<br>CAGAGACCTTTATCCAGTGCTTTGGGGGACACAGAGGATTGAGGGGCAGAAGCGGG<br>GAGAAGGTCGGAGACATCGTAGATTTTTCTTCAGTTCAGCATATCAAAGTGCTATATTT<br>TGGGGTGTCTGTTTCTGAGTCCTAATACCCATGTTGAGGTGCAGAGCCAAAGATTCTT<br>ACTGGGGGCTTTTCTCATAGGTATACTCTGCTTGCTCAGCCAGCCACAACCAGCAACA<br>TTGCAGACTCTCAGAAGGAAAGCAAGTATTCTCCATAAATCACACTGTTTGTACAGTC<br>TAGGCAGGCCGATACAACACAATTCAGTGCACCAGGTGGGCAAGGTCCTTATCAGTTA<br>GTGACTTGGGAACCTTCCAAAAGCCAAGTTCCCAGGTGCTAGCCATGGGCCAGCCCC<br>ATAAGCCGGCCCTTCCGTATTGGGGC

Junction VII	J8/J12	AATATATATTACTAATTTGCATTAGTAATATATATTACTAATTTGCATTAGTAATATATTAGT<br>GTTATAAATATTTTTAGTAATATATGTACTAATATTTTTGTATAGTAATAAATACCACTAAA<br>TGTTTATATTGCTGATACTAGTAAAATTGGAGAAGTTTTATGTATATTTATGTACATTTTA<br>CGTATTTTTTTCTTAGTTACATTTTTAGGTTTTGCAAATCCAGAATTTTTATCCCATCTGA<br>TTATTAATGTATGACAATAATTATGTCAATAATTATGACAATAAGATTGTCTGATAACATT<br>TTCTTAAAGAATGAATCTTTTACCTTAGCATACATGTGATTCTATCGAGACACTGCAAG<br>GATACTAGCGACTGTCCCATAGGGGCACACAGGTGTTTCATGTTGTTGCTTTCTACTTC<br>TGATAACAGATCATGAAGCTATGTCATGACATGCCCTCTAGGCTTTTGGTAGCTGATAT<br>TTTCAAATATCAGAGTTTTGTATTCGCCCTCAGATTTCAACAGATTTCAGCAAGATACC<br>AAGTCAGCTCCAACAAATGTGGAAAAACAATCTTTACTAATAGTA▲AAACTCGAATT<br>TTAACTTTATTGGGTCAAGACACAATAAAAGTTTTTCCTTGCAAATTCTGACACGGTT<br>GGGTGATTCATTCCAAGTAGTGTCTTGATTATCCAGGTTCTGTGCATTCAGACAATCTT<br>CTGTT▲GTTCGATGACTTACGGGAGGCACCCAGAACAGGTGGGTGTAATTCAGCGTG<br>ATTACAAGCCACCTATGAAAGAGTGACACATACATTTCCGTGGGAATAAACAGAAAA<br>AAAATCAGTAATGTCAGCAGAGTCTGTGTCTTTCCTAATGCCCACTCTAACCTAAAGG<br>ACAGGCAAGCAGGAGATGG▲TACAACTCTGCCTCCCAGTTTCAAGTGACTCTTGTGC<br>CTAAGCCTCCCAAGTAGCTAGGATTACAGGCGTGTGCCACCATGCCTGGCTAATCTTT<br>▲CCTAATATACCAATAAATATTTCTTTCCTTCCTTT▲CATGCCTGGCCTTGAAATTAT▲<br>ATTATATTTCTTATCTATACTGACTGAAATTTTACTGAAACTTCTAAGCCAAACTCAGAT<br>GCCACCTCTCTCTTGAAGACTTTTGCTGGTCTGCTTTGTCATCCTTACGTGGTTCATAG<br>CACTCTGCCTTCTGTTACTATTA▲TCCTGGATAATATCCTGCAGAGTGTTTTCCATCTC<br>AAAAAAA▲AAACTTCAGTAGTTGGTTGGGCATTTTATTGAGTAGAGGTGACTCCATG<br>CAGTTCTTTTTGGGGGAAGGATGCTGCACATTCCAGGGTTTAACAAAAATT▲GCTGT<br>CCTGAAGCAGGTGTTCAGG▲CCAATTCTGATTCTATTATGTAATTTCATTATGACACAG<br>GGTGTTTGGTTTGCTCCTTTACCAAATACAGGGTTAGCTACCAAATATGGGGTTAGTTA<br>AAGAAGGGAGATGGGGTCCTCAGATCTGTGTAATAGGTTTTATATCTGTGTGATAAATT<br>TCTAAAACCTTGAGATGTAAAGAATCTGTATGAATAAAGAGTGGGATATACATGGGGA<br>▲CCAACCTCACATACAAC▲GTGTAGTGCACGCTTGATAGGTGAAGAAGCAAATACT<br>CATTTAAAATAAGAGGTCTAATTCTTCCTGTTGAAAACAAAGAGA▲AAAAATTACTG<br>AAAGAGTAGATGTTAGATGCCCTTCCCACAAAATGTAACCATGTGATGGGTATGCTAA<br>TTTG▲ATTATACAATTAATAA▲AGATGTAAAGTAACTTACTCAAAATCACCCAGCTAC<br>TGACAGGCAAATCTGGGCCCAGAGCCCATGCTCTAATCTAAATCATTACTACCTCCCT<br>GCTGGTAACAAACAA▲CTACATCTCCTCTGGCCTTTCTATTCCTTGAGTCAGAAAAA<br>AATATTGAAATTACGTGAATTAATAATCTTACAATG▲TCACTTTAAGTAAAAATCAGAT<br>GTAACTAACAAAGCACTGGTGTGATCTCAGGGCTTAGAAACTAGTGCTGATCATTATT<br>CTAGAGAGTTACTACAATGTTCTTTCCAAAGTCAGCAAAACCAGAATAAATTAGCAGC<br>TTCCTCAGGTGGTGGTTTTGAAAATCTACACTCACTTGATATAAGTTCTGGTATGTTTA<br>TTTATAAACGTCTTATTACTTTAAATTATCGCCTCATGGAA▲AAACCCTAGGCAATACC<br>AGTCAGGACATAGGCATGGGCAAGGATGTCATGACTAA▲TATTTTAGGACTA▲AAAG<br>TTTTAGTAGTCTGGATAGATCAAACTAGCCACAACATTCCCTTAAGTCAAAGGCTAATT<br>CAGAGCAAGCCCCTAACTCTCTTCAATTCTGTG▲TTTTCACTGTTCCCTAACTCCTAC<br>AGCAGCACATCC▲CAAGACACATAATCATCAACAACTCTAAAAAGTGAAGCTTTTAA<br>TCTAAAATTAAAATTTTATATGTTTTAAAATTTTATATAAAATTAAATTTTTAAAACATAT<br>GTTTTAAAACAAGAAATTTTAAAGTAACATATATAATTTAAATA▲TGTGGAAGAAGAA<br>ATAGACACCCTATCTGTATACATTAGTC▲CTTTCTGACT▲CCCTTACCCAAAGCAGAG<br>TTGATTCCCCCCTCCCCTGGACGGC▲GTAATCCCAGCACTTTGGGAG▲GCCAA▲GG<br>CGAGCGCCTGTAGTCCCAGCTACTCAGGAGGCTGAGGCAGGAGAATGGCGTGAACCC<br>GGAAGTGGAGGTTGCAGTGAGCCAAGATCATGCCACTGCACTCCAGCCTGGGGACA<br>GAGCAAGACTCCGTCTCAAAGAAAAAAGGAATTGTTGACTGGAATAGTGGGTGATTA<br>ATTCCAGCTACACAGACAGTGTTCACGTGCTAGCTCCTAGCACAGTGCTAGAAGCCA<br>GAAATCATATCAAACATGAAAGACACAATCCTGACTTCATGGAGCTTTCAGGCTGTAC<br>TAAGATTTCCAGAGAAGCAGGACATGTCCATAAGCGAGGACATTTGTTGATAACCAGG<br>TTGCCCTGGCGGGCTGGCAGAAGCTGAGGAACAGGAGGAAAGGTTGGACCACATAG<br>AATGTGAGAGGCCCCAGTACGTCAGCACACAAGAAACCTTGGAAATAATCTCGTTCA<br>ATGTCCTCACTGAAGAGCTGAGAAGTAAGGCCTGGAGAAGTAAAGTGACTTGCCCAA<br>GATCACCCAGCGAAGACCTGCAGATGCAGAGT

Junction VIII	J10/J7	TGCCACGAGAACAGCAAGTTCCAGATGGGGCTGCTCCTTCAGCCCGGATCCTAGAAT<br>GATGATGCATAGATCGCAGTTGTCATGTAATATAACTGAGAAATATACCTTTGGGCTCAT<br>AAGCTATTGAGATTTTGAGGTTTGAACAGCAGCATAACCAAACAGAAGATATACACCC<br>CAGCTGTGATAAATACACACTCTTGCTGTAACCTGTATCCTGCGGGTTAGATAATAAAT<br>CCTCTTTTGTTTTAGTAGTGGTTGGTTAGAACTTTACTTTTTGCTTTCTACAAACCAATA<br>AAAAGGAAAATTCAACCAATATGGTCTCAAAGA▲AATTTGTTTGAGTTCATTGTAGAT<br>TCTGGATCTTAGCCCTTTCTCAGATGAGTAAGTTGCGAAAATTTTCTCCCATTTTGTAG<br>GTTACCTGTTCACTCTGACAGTAGTTTCTTTTGCTGTGCAGAAGCTCTTTAGTTTAATT<br>AGATCCCATTTGTCAATTTTGGCTTTTGTTGCCATTGCTTTTGGTGTTTTAGACATGAA<br>GTCCTTGCCCATGCCTATGTCATGAATGGTAATGCCTAGGTTTTCTTCTAGGGTTTTT▲<br>CTTCTGGGTGTTGCCATGGCATTGGTAAACTGACATGGCACTGATGGGCGTGTCTTAT<br>GGAGAGGGGCTTTCACATTTTCCCCGTTTCAGCTAGTCTTCAGTCTGGTCCGAAGTTT<br>GAGTACCCCCCTCCAGAGTTGCATCCCACCTTCTACCTCTATCTGACCCATTTTTCATA<br>TCAATTTGGGCCCCACCGACAAGTCTTTATTTTAAAGCACAAGCCTCCTCTTCTAACT<br>CTTCCCAGGTGTGAGGCCCATTGAGATGCACTGCTCAGATTTCCTTCAAGAGAATGTG<br>CCGTAGGGAGCATAGTGGGTCAACAGCCCCAGTGGCAGCCCCATGAGCTGAGGGTGT<br>GTTTTCTGCAGGTTGCTCCCAGCCCATGACAGCT▲AGTTCAGTGTAGGTTGTGCAAC<br>TGGCAACAGAAACTGGCATGAGATGAAGGGGTATAATCCATGCTGGTCATCATTAGTA<br>TGTGTGATTTTTAAATAGGACAGAGGCAATATACT▲AAAATGCTCATCATCACTGGCC<br>ATCAGAGAAATGCAAATCAAAACCACAATGAGATACCATCTCACACCAGTTAGAATG<br>GAGATCATTAAAAAGTCAGGAAACAACAGGTGCTGGAGAGGATGTGGAGAAATAGG<br>AACACTTTTACACTGTTGGTGGGACTGTAAACTAGTTCAACCATTGTGGAAGTCAGTG<br>TGGCGATTCCTCAGGGATCTAGAACTAGTAATACCATTTGACCCAGCCATCCCATTACT<br>GGGTATATACCCAAAGGATTATAAATCATGCTGCTATAAAGACACATGCACACGTATGT<br>TTATTGTGGCACTATTCACAATAGCAAAGACTTGGAACCAACCCAAATGTCCAACAAT<br>GATAGACTGGATTAAGAAAATGTGGCACATATACACCATGGAATACTATGAAGCCATA<br>AAAAAC▲TTGATTGGATCCTGCTCAATTTGGGATACCCCAATGAGCAGCTTTGGCTCT<br>CATTGGCTTTCATTTGGCTCTGGGACTCCCCATCCATGTGGTTGAAACTTTCTCTGAAC<br>TGCTGCCTGAGCCTCTTCCTCCCCAGTCTTTCCTTCTTTCACAGATGTGAGACAA▲CT<br>GACACCTGCAAGGCCATGGACTCCTAACTGACCTATAGTCACTGGCAGTGTCCCTGG<br>CACGTAGCACAGTGCCTGGTACCTAGATAAACCTTAACATAAACTAACATAAACTCCT<br>AGGGATTTAAGAAGCAATTAATTGGACAAGAGAATCAGCACCACTCTAAGACAAGAA<br>ATAAGTAGTTATTTTAGGCCGGGCACAGTGGCTCACGCCTGTAATCCCAGCACTTTGG<br>AGGCTGAGGTGGGAGGATCACCGGAGGTCGGGAGTTCAAGAGCAGCCTGG▲CCAC<br>AGCCAGTGTCTCCTCCACTAGATCCGAGCCAGTGGCTCTGCCCAGGTGG▲ACAGTGA<br>TTCAGTAGAAATCACAAGCAACAGGTTTGTTGTGCTCAAATCTTCATGTCTTTGCTGG<br>AGTTTTTGTTGCATTTTGCTGATAATTACTAAGGAACACGGCATTTCTAGTAATTTTTTT<br>CAATTACAAAAGAAAAAAACAAAAAAGGTA▲GTATGTATCTTCACAATCCATGTTCT<br>TCTG▲CCATTGTCTTGGGGATTAACATTCAGCTCCTCGTTACTTATGCAAGTTTCTACA<br>GCCAATTTAGATTCCTCCTCAGAAAATTAAATTTTCTTTTCTTTCACATTGTCAGGCTG<br>CAAATTTTCCAAACTTTTATGCTCTGCTTTC▲CAGTTTTGTCGAGGCAATGGAATTATA<br>GCTCTGTGCCTGCTCCTGCAAGCAGGGCTACCCCATGGGCAGGGCTACCCCACAGGC<br>AGTGTGTCAAGAGGAGCAGCTCAGGGGCATTTCTGTAGTCATAACCACTTTTAGTAAC<br>AAGCAAATTAAGGGGCAGATTATGCAGAAATTTCT▲TGGGCCGTCTGCAGGCTGAGG<br>AGCAAGGAGAGTCAGTCTGAGTTCCAAAACTGAAGAATTTGGAGTCTGATGTTTGAG<br>GGCAGGAAGCATCCAGCATGGGAGAAAGATGTAGGCTAGGAGGCTAGGCCAGTCTC<br>CCTTTTCACATTTTTCTGCCTGCTTAAATTCTAGCCACACTGGCAGCTGATTAGACTGT<br>GCCCACCCAGATTAAGGGTGAGTCTGCCTTTCCCAGCCCACTGACTCAAATGTTAATC<br>TCCTTCCGCAACACCCTCACAGACACACCCAGGATCAATAGTTTGTATCCTTCAATCC<br>AATCAAGTTGACACTCAATATTAACTATCACAATATGCTTATAATTGATTATTACCAATG<br>GCCAAGGTAACCTTCTACTCAGTTTTTGCCTGTACACTAACTTGTCCAAGTCAGCGAT<br>CGTGAACCTGACCCACCAAAAAAAACAGTTAACATGAAGGCATTTGGAAATGATAAT<br>ACTCAGAATTAATGCAAGTGTATAAAAGTGGTCACTCATAAAATAGTGCTACTAAGAA<br>CATAAACGAATAGAAGAATTGCTCATCTCTGCATTATTTTATCCCATAAAATAATCAGA<br>GACATGTACAAGTATTATGCAAAATGGCATTCACCTTTTGTTAGTGTTATAGGAGGTTA<br>GCCCTGCTGAAGCCTGAGC▲GAGTGTTTTCAGATGTGTCAATTCTACAGGAAAAATT<br>AGGTTGACTGACTGGGAAGAGCAAACATCTGAGATATTTAAATGATGTACAAAACTC<br>ACCTATTGTCGCTATGCAGACAAGAAAGTTTGAGTGAACAGATAAAAACTTTCTGACT<br>GATTCAATACCTGCTT▲GCAATTGTGTGGAAAGGCCGTGAGCTGAAGTCTTGGCTCC<br>ATGTTATCC▲CAGTTATCTACAAAGATATCAGGTAATTTCGGTATTTGGGATACAGGTA<br>AAAGAGGTTTCTTGTTTCTTTACATTTACTATTGAAGAATAAAGTTAAAGAACCACAA<br>AATGTATTGTTAAAAGGGATTTTAAAGATCATCTCATCCAACTTCTTCAGTTTACACAT<br>GAAGAAATCAAGGCCCAGAAAAGAGAACTGGTCTACAC

The micro homologies and insertions are shaded grey. In Junction VI, Junction VII and Junction VIII, ‘▲’ means the DNA breakpoint.

Supplementary Table S4: Chromosome position of inserted DNA fragments in Junction VII and Junction VIII

Junction Name	Insertion Name	Size	Chr	Strand	Amplified regions	Chromosome position Stare	Chromosome position End
Junction VII	Ins1	133	Chr8	+	non-amplified	116032454	116032573
	Ins2	185	Chr8	+	non-amplified	123094127	123094311
	Ins3	95	Chr8	-	non-amplified	129004478	129004572
	Ins4	35	Chr8	+	non-amplified	123048272	123048306
	Ins5	21	Chr8	+	non-amplified	123051956	123051976
	Ins6	142	Chr8	+	non-amplified	123051972	123052113
	Ins7	42	Uncertain	Uncertain	Uncertain	Uncertain	Uncertain
	Ins8	100	Chr8	+	non-amplified	128946414	128946513
	Ins9	24	Chr8	+	non-amplified	123093075	123093097
	Ins10	215	Chr8	-	non-amplified	116437909	116438123
	Ins11	17	Uncertain	Uncertain	Uncertain	Uncertain	Uncertain
	Ins12	82	Chr8	+	non-amplified	123278744	123278825
	Ins13	73	Chr8	+	non-amplified	122476049	122476122
	Ins14	16	Uncertain	Uncertain	Uncertain	Uncertain	Uncertain
	Ins15	109	Chr8	+	non-amplified	129056716	129056824
	Ins16	77	Chr8	+	AmpH3c	128487159	128487232
	Ins17	238	Chr8	+	non-amplified	116447832	116448069
	Ins18	55	Chr8	-	non-amplified	116035344	116035398
	Ins19	13	Uncertain	Uncertain	Uncertain	Uncertain	Uncertain
	Ins20	96	Chr8	+	AmpH3c	128487557	128487652
	Ins21	36	Chr8	-	non-amplified	124161730	124161765
	Ins22	149	Uncertain	Uncertain	Uncertain	Uncertain	Uncertain
	Ins23	41	Chr8	-	non-amplified	123093155	123093581
	Ins24	10	Uncertain	Uncertain	Uncertain	Uncertain	Uncertain
	Ins25	43	Chr8	+	non-amplified	129002421	129002463
	Ins26	21	Chr8	-	non-amplified	128892797	128892816
	Ins27	5	Uncertain	Uncertain	Uncertain	Uncertain	Uncertain
Junction VIII	Ins28	259	Chr8	+	AmpH2d	122063797	122064055
	Ins29	382	Chr8	+	AmpH3a	126716332	126716713
	Ins30	115	Chr8	+	AmpH3a	126187144	126187258
	Ins31	433	Chr8	-	AmpH2d	122063266	122063698
	Ins32	165	Chr8	+	AmpH3a	126716721	126716888
	Ins33	282	Chr8	-	AmpH3a	126657926	126658197
	Ins34	53	Chr10	+	AmpH4b	123494405	123494457
	Ins35	155	Chr8	-	AmpH2d	122022909	122023063
	Ins36	30	Uncertain	Uncertain	Uncertain	Uncertain	Uncertain
	Ins37	144	Chr8	-	AmpH3b	126724123	126724266
	Ins38	177	Chr8	+	AmpH3a	126716139	126716315
	Ins39	679	Chr8	-	AmpH3c	126824455	126825133
	Ins40	168	Chr8	-	AmpH3c	126828618	126828785
	Ins41	49	Chr8	+	AmpH3a	125942770	125942818

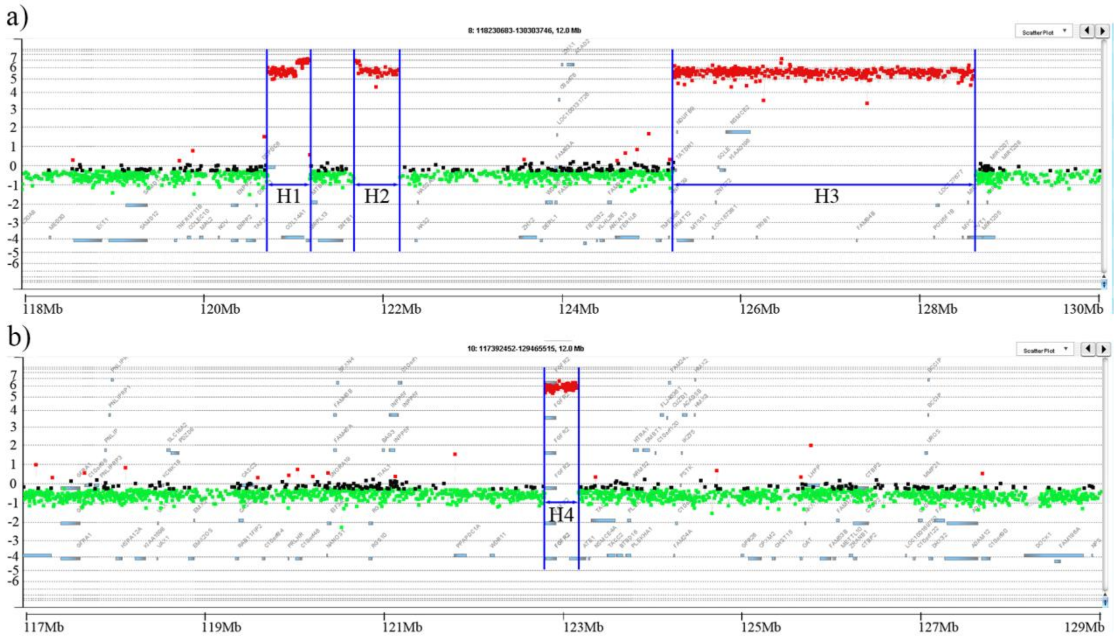

Supplementary Figure S1: The amplicons identified by Agilent human genome CGH microarray (2×400K). Three discontinuous amplicons originated from 8q24.12-21 (A) and one amplicon from 10q26.13 (B). X axis represented chromosome coordinates. Y axis represented log2 ratios of the copy number normalized by normal controls, showing distinct sub-regions with different overall copy numbers. Blue vertical lines depict boundary positions for each amplicon.

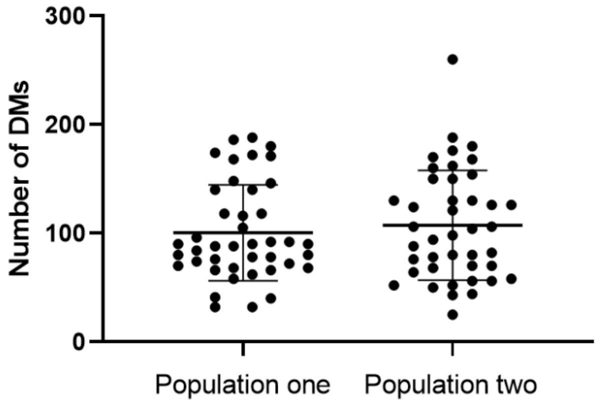

Supplementary Figure S2: The numbers of DMs in two populations in NCI-H716 cells.
